# Complement activation in immunological neurological disorders: mechanisms and therapeutic strategies

**DOI:** 10.3389/fneur.2025.1695461

**Published:** 2025-12-02

**Authors:** Jin Y. Chen, Nirav Sanghani, Kaitlyn Palmer, Yuebing Li, Feng Lin, Justin R. Abbatemarco

**Affiliations:** 1 Department of Immunity and Inflammation, Lerner Research Institute, Cleveland Clinic, Cleveland, OH, United States; 2 St. Francis Hospital and Medical Center, University of Connecticut School of Medicine, Hartford, CT, United States; 3 Mellen Center for Multiple Sclerosis, Cleveland Clinic, Cleveland, OH, United States; 4 Neuromuscular Center, Cleveland Clinic, Cleveland, OH, United States

**Keywords:** complement, innate immunity, myasthenia gravis, aquaporin-4 IgG-positive neuromyelitis optica spectrum disorder, complement-targeted therapies

## Abstract

Recent advances in the understanding of immune-mediated neurological disorders have led to a paradigm shift toward pathophysiology-directed therapies. Central to this progress is a deeper appreciation of the complement system, a key component of innate immunity, and its role in neuroinflammation. Complement activation, while essential for host defense and tissue homeostasis, has been implicated increasingly in a spectrum of central and peripheral neurological disorders where complement dysregulation contributes to inflammation, cellular damage, and disease progression. Breakthroughs in conditions such as myasthenia gravis and aquaporin-4 IgG-positive neuromyelitis optica spectrum disorder underscore the therapeutic potential of targeting complement pathways to improve patient outcomes. In this review, we provide a comprehensive overview of complement activation pathways, regulatory controls, and their involvement in various autoimmune neurological diseases. We also highlight current and emerging complement-targeted therapies, many of which are now completing or entering clinical trials. Together, these insights offer a holistic perspective on the complement system as both a contributor to and a target for intervention in neurological diseases.

## 1 Introduction

Over the past several years, our understanding of immune-mediated neurological disorders has evolved dramatically, catalyzing a new era of pathophysiology-directed therapies. Advances in complement-targeted therapeutics have been particularly striking in diseases such as myasthenia gravis (MG) and aquaporin-4 IgG-positive neuromyelitis optica spectrum disorder (AQP4 + NMOSD). These diseases now serve as a blueprint for the therapeutic potential of complement modulation to yield significant improvements in function and quality of life.

The complement system is an important component of innate immunity and consists of a cascade of proteolytic events, collectively defined as complement activation, that lead to the opsonization of pathogens, recruitment of immune cells, and direct lysis of target cells (1, 2). While its primary function is to protect the host from infections and to clear apoptotic cells, dysregulation of the complement system has been implicated in a range of neuroinflammatory and autoimmune neurological disorders, where excessive or misdirected complement activation contributes to inflammation, cellular damage, and progressive neurological impairment.

This review provides a comprehensive view of complement systems in selected autoimmune neurological diseases. We examine complement activation pathways, regulatory mechanisms, and pathological roles in various nervous system disorders. We highlight the most promising complement-targeted therapies—ranging from monoclonal antibodies to fusion proteins and cyclic peptides—many of which have already gained regulatory approval or are currently in pivotal clinical trials. By providing a holistic view of complement biology in the nervous system, we aim to clarify its role as both a mediator of disease and a therapeutic target in neurological disorders.

## 2 Overview of the complement system

### 2.1 Three complement activation pathways

**Figure 1:**
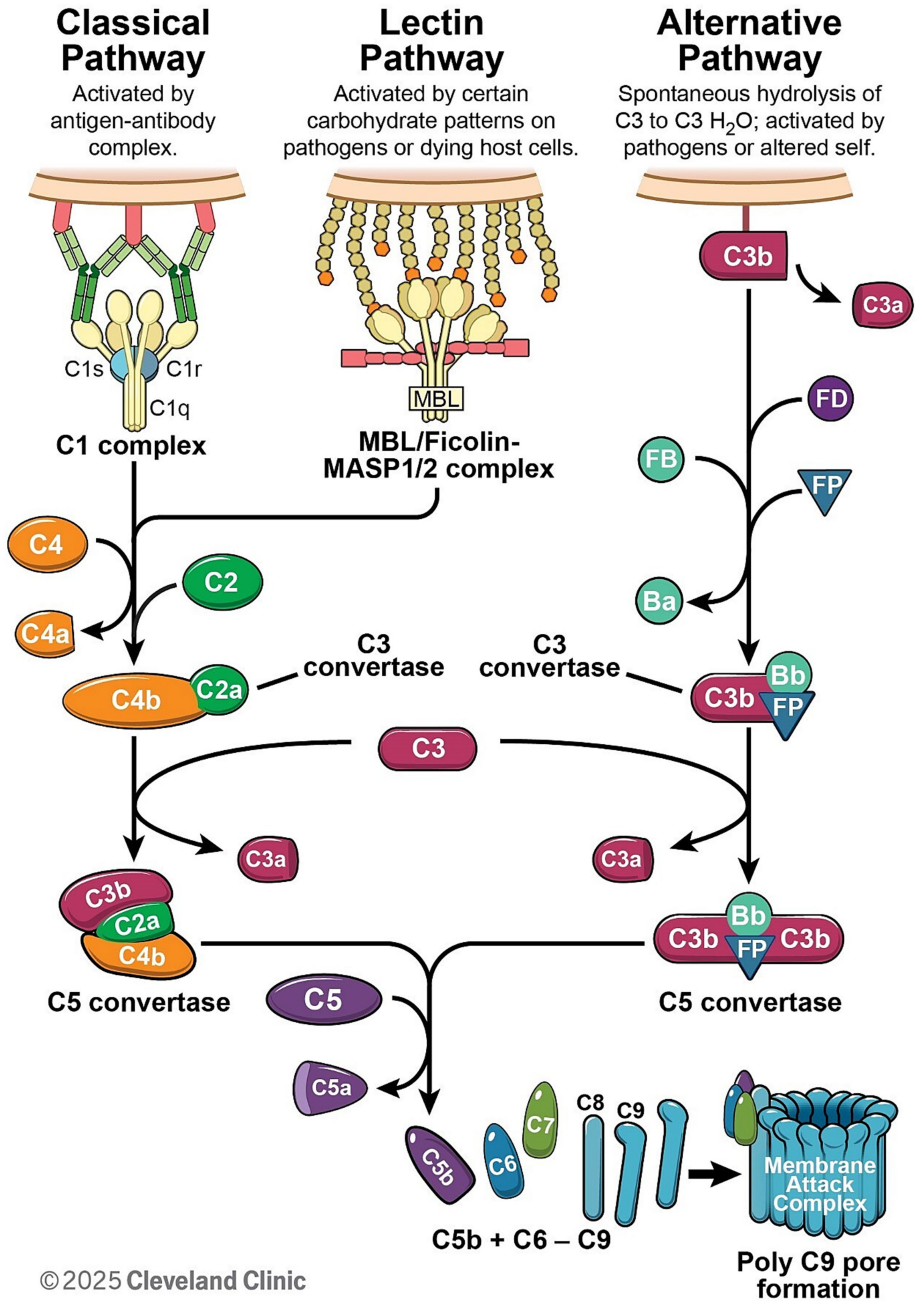
The complement cascade can be initiated through one of three pathways: the classical, lectin, or alternative pathway. The classical pathway is initiated when the C1 complex binds to antibody–antigen complexes, whereas the lectin pathway is activated through the recognition of pathogen-associated molecular patterns. Both pathways culminate in the formation of the C3 convertase C4b2a. In contrast, the alternative pathway is spontaneously activated and forms the C3 convertase C3bBb with the assistance of Factor B (FB) and Factor D (FD), and its stability is further enhanced by properdin (FP). All three pathways converge at the cleavage of complement component C3. The cleavage of C5 facilitates the formation of membrane attack complex.

Complement activation proceeds through three distinct pathways: classical, lectin, and the alternative pathways (Figure 1). The primary distinction among these pathways lies in their initiation mechanisms. The classical and lectin pathways require specific pattern recognition molecules for their initiation. In contrast, the alternative pathway does not depend on external recognition but instead perpetuates its activity through spontaneous hydrolysis and self-amplification. Once activated, these pathways converge on a shared cascade involving the cleavage and activation of several complement proteins, many of which are produced by various cell types. Although the majority of complement proteins are synthesized by hepatocytes (3–5), other cell types, including monocytes (6), fibroblasts (7), endothelial cells (8), epithelial cells (9), and glial cells (10, 11), are also capable of producing complement components. Most complement proteins function as acute-phase proteins, and their expression can be upregulated by pro-inflammatory cytokines (8, 12), enabling a rapid response during acute inflammatory states (13, 14).

The classical pathway (CP) is initiated by antigen–antibody immune complexes (Figure 1). C1q is the recognition molecule that binds to the Fc portion of IgG or IgM within the immune complexes and triggers CP activation. C1q binding triggers activation of the C1 complex (C1qC1r_2_C1s_2_), which then cleaves downstream C4 and C2 to generate the CP C3 convertase C4b2a, leading to C3 cleavage (Figure 1). Notably, complement recruitment and activation is highly subclass dependent and is determined mainly by their affinity for C1q, which follows the order IgG3 > IgG1 > IgG2 > IgG4 (15). The hinge also regulates the Fab–Fc flexibility, which is IgG3 > IgG1 > IgG4 > IgG2 (16) and, in turn, affects immune complex formation and C1q engagement (17). As a result, IgG1 and IgG3 are potent activators of complement, IgG2 is weak, and IgG4 has no or little capacity to activate complement.

The lectin pathway is triggered by mannose-binding lectin (MBL) or ficolins binding to pathogen-associated molecular patterns (Figure 1). For example, MBL recognizes specific carbohydrate patterns that are rarely present on host cells but are expressed frequently on pathogens and dying cells (18). Ficolins bind to acetyl groups such as N-acetylglucosamine (GlcNAc) and N-acetylgalactosamine (GalNAc) found on the surface of bacteria (19, 20). As a result, MBL/Ficolins activate MASP1 or MASP2 in the blood, resulting in cleavage of C4 and C2 and assembly of the C3 convertase C4b2a, which promotes cleavage of C3 (Figure 1).

Uniquely, the alternative pathway is spontaneously activated via low-level hydrolysis of C3 into C3(H_2_O) (so called complement “tickover”), which occurs naturally in the blood at a low rate. The hydrolysis of C3 to C3(H_2_O) can be accelerated through interactions with various biological and artificial interfaces, such as biomaterial surfaces, lipid membranes, and molecular complexes (21). A low level of spontaneous activation of C3 also allows Factor B and Factor D to form a C3 convertase C3(H_2_O)Bb, which cleaves further C3 into active fragments C3a and C3b. C3b can then bind to local cell surfaces and amplify the cascade through the AP convertase C3bBb (Figure 1). MASP-1/3 have also been proposed to have a role in regulating basal Factor D activity (22, 23). More importantly, the alternative pathway serves as the primary amplification mechanism within the complement system, amplifying immune responses via C3b generation. This amplification can be initiated not only by the alternative pathway itself, but also through C3b produced via the classical or lectin pathways.

### 2.2 Consequences of initial complement activation

Upon activation, the classical and lectin pathways generate C4b2a convertase, whereas the alternative pathway forms C3bBb convertase (Figure 1). These convertases cleave C3, a plasma protein of high concentration (24), leading to the formation of C5 convertase (C4b2aC3b for classical and lectin pathway, and C3bBbC3b for alternative pathway). These C5 convertases drive the assembly of the membrane attack complex (MAC; C5b-9), which disrupts cellular membranes, altering ion homeostasis (Figure 1). MAC is widely recognized as a potent driver of cellular function disruptions, inflammatory responses, and progressive tissue damage, such as demyelination (25–27) and axon degeneration in neurons (28, 29).

During complement activation, potent anaphylatoxins such as C3a and C5a are released during C3 and C5 cleavage (Figure 1), also triggering inflammatory responses via C3a or C5a receptors, which are G protein-coupled receptors. These receptors are widely expressed on cells of the myeloid lineage, such as neutrophils, basophils, eosinophils, mast cells, macrophages and microglia (30), and are also found on astrocytes (30), neurons (31), and endothelial cells (32, 33). In central nervous system (CNS) disorders, C3a- and C5a-mediated signaling promotes microglial activation (34), leading to phagocytosis of synapses (as seen in neurodegenerative diseases) and destruction of oligodendrocytes (35). In peripheral nervous system (PNS) diseases such as Guillain-Barre syndrome (GBS), antibodies against gangliosides activate the complement system. The resultant complement deposition on the surface of Schwann cells and axons leads not only to direct injury, but also to recruitment of macrophages. These macrophages then invade the myelin and axons, causing further injury and propagating the immune response (29). Although C4a is also produced during complement activation, its anaphylatoxin activity is significantly weaker than that of C3a and C5a (36) and is less studied in neurological disorders.

## 3 Regulation of complement activation and dysregulation implications

Complement activation is tightly regulated by regulatory proteins to prevent excessive activation that may result damage of healthy host tissues. Deficiencies or mutations in these regulatory proteins can result in uncontrolled complement activity, contributing to the pathogenesis of various neuroinflammatory diseases.

### 3.1 Regulation at the initiation phase

At the initiation of the complement cascade, C1 esterase inhibitor (C1-INH) is a key regulator of the classical pathway. C1-INH inactivates C1r and C1s by forming covalent complexes (37). While C1-INH deficiency is associated classically with hereditary angioedema (38), no neurological disorders have been reported in association with C1-INH deficiency (39).

For the lectin pathway, regulatory proteins including Map44 (40) and Map19 (41) inhibit the binding of MASP1 and MASP2 to MBL and ficolins. While the role of these regulatory proteins in neurological disorders remains under investigation, lectin pathway dysregulation has been implicated in cerebral ischemia and neurovascular inflammation (42).

### 3.2 Regulation at the activation phase

Complement activation is tightly regulated by a variety of proteins that function either in the fluid phase or on cell surfaces. Key fluid-phase regulators include C4-binding protein, Factor H (FH) and Factor H elated proteins, Factor I, clusterin, and vitronectin. Factor I inactivates C3b and C4b, and its deficiency has been associated with fulminant CNS disorder involving the brain and spine cord because of unchecked complement overactivation (43, 44). Clusterin and vitronectin act downstream to inhibit MAC formation on host membranes. Clusterin modulates amyloid beta aggregation and clearance in the brain (45). Genetic studies have identified clusterin as a major risk factor for Alzheimer’s disease, linking complement dysregulation to neurodegeneration (46).

On the cell surface, complement regulatory proteins such as membrane cofactor protein (MCP; CD46), decay-accelerating factor (DAF; CD55), and complement receptor 1 (CR1; CD35) prevent excessive complement activation by reducing the C3/C5 convertases activity. CD59 (protectin) prevents C9 from polymerizing and inhibits MAC assembly. Mutations in CD59 are associated with a rare inherited form of demyelinating neuropathy (47, 48). Notably, AQP4 is expressed on many peripheral organs (i.e., kidney, stomach, skeletal muscle) that are exposed to circulating AQP4-IgG. These tissues are relatively spared in AQP4 + NMOSD. The CNS-selective vulnerability may be attributed to the limited expression of complement regulatory proteins (CD46, CD55, and CD59) in the CNS, which renders astrocytes particularly susceptible to AQP4-IgG–induced complement-mediated damage (49, 50).

### 3.3 Regulation on effectors

Complement effector functions are also regulated to limit inflammation within the nervous system. Carboxypeptidase N inactivates the potent inflammatory mediators C3a and C5a by converting them into desArg forms (51). This enzymatic conversion limits prolonged inflammation, helps maintain homeostasis, and is particularly important in the context of chronic or relapsing neuroinflammatory disorders.

## 4 Current complement-targeted therapies

Recent advances in complement-targeted therapies have revolutionized the treatment of many complement-mediated diseases. These therapies aim to inhibit specific pathways or complement effectors, either by supplementing complement regulation, or inhibiting effector functions in one or multiple pathways. This review focuses on the inhibition of complement activation.

### 4.1 Overview of complement-targeted therapeutics across pathways

Therapeutic strategies targeting the complement system have offered multiple points of intervention across its activation pathways. Table 1 summarizes key complement targeting drugs in clinical use or development, their mechanisms of action, targeted pathways, and clinical indications. The specific use/development of these drugs in PNS and CNS disorders will be discussed in later sections.

**Table 1:** Overview of current complement targeted therapies.

Pathway targeted	Target	Agent	Modality	Indication/use
All pathways	C3	Pegcetacoplan	Peptide (PEGylated)	FDA approved for PNH and AMD, being evaluated in C3 Glomerulopathy and IC-MPGN (NCT05809531).
		ARO-C3	RNAi therapeutic	Being evaluated in complement-mediated renal diseases (NCT05083364)
Classical pathway	C1 esterase inhibitor	Cinryze	Plasma-derived protein	FDA approved for Hereditary angioedema
	C1s	Sutimlimab	Monoclonal antibody	FDA approved for Cold agglutinin disease
		Riliprubart	Monoclonal antibody	Improved C1s targeting
	C1q	Tanruprubart (ANX005)	Monoclonal antibody	Being evaluated in Huntington’s disease (NCT04514367), Autoimmune Hemolytic Anemia (NCT04691570), Amyotrophic Lateral Sclerosis (NCT04569435), GBS (NCT04035135, NCT04701164, NCT07020819)
Lectin pathway	MASP2	Narsoplimab	Monoclonal antibody	Being evaluated in HSCT-TMA (NCT05855083)
Classical and lectin pathway	C2	Empasiprubart (ARGX-117)	Monoclonal antibody	Being evaluated in Multifocal motor neuropathy (NCT06742190), Dermatomyositis (NCT06284954)
		Nab1B10	Nanobody	
Alternative pathway	Factor B	Iptacopan	Small molecule	FDA approved for PNH, IgA nephropathy, and Membranoproliferative glomerulonephritis
	Factor D	Danicopan	Small molecule	FDA approved for PNH (adjunct to Eculizumab)
		Vemircopan	Small molecule	Evaluated in PNH (NCT04170023), and Proliferative Lupus Nephritis and Immunoglobulin A Nephropathy (IgAN) (NCT05097989)
		Lampalizumab	Monoclonal antibody	Evaluated in Geographic atrophy (AMD) but failed
	MASP3	OMS906	Monoclonal antibody	Being evaluated in PNH (NCT06298955) and C3 Glomerulopathy (NCT06209736)
Terminal pathway	C5	Eculizumab/Ravulizumab	Monoclonal antibody	FDA approved for PNH, aHUS, gMG, and AQP4 + NMOSD
		Crovalimab	Monoclonal antibody	FDA approved for PNH, being evaluated in aHUS (NCT04861259, NCT04958265) and sickle cell anemia (NCT04912869, NCT05075824)
		Cemdisiran	siRNA	Being evaluated in PNH (NCT05744921) and gMG (NCT05070858), Sporadic Inclusion Body Myositis Conditions (NCT06479863), Geographic Atrophy (NCT06541704)
		Pozelimab	Monoclonal antibody	Being evaluated in PNH (NCT05744921) and gMG (NCT05070858), Sporadic Inclusion Body Myositis Conditions (NCT06479863), Geographic Atrophy (NCT06541704)
		Avacincaptad pegol	RNA aptamer	FDA approved for geographic atrophy
		Zilucoplan	Peptide inhibitor	FDA approved for gMG
		Nomacopan	Peptide inhibitor	Being evaluated in Bullous Pemphigoid
	C6	C6 inhibitors	Monoclonal antibody	
	C7	C7 inhibitors	Monoclonal antibody	
C5aR-C5a signaling	C5aR1	Avacopan	Small molecule	FDA approved for ANCA-associated vasculitis
		ACT-1014-6470	Small molecule	
		ALS-205 (PMX-205)	Small molecule	
		Avdoralimab	Monoclonal antibody	Being evaluated in severe COVID and Bullous Pemphigoid
	C5a	Vilobelimab	Monoclonal antibody	FDA approved for emergency use, being evaluated in ARDS (NCT06701682; NCT06703073) and Pyoderma gangrenosum (NCT05964413)

Inhibiting C3 and C5—central nodes of the complement cascade—remains a cornerstone. C5 inhibitors, such as eculizumab and ravulizumab (52, 53), block C5 cleavage and prevent MAC formation, with ravulizumab offering extended half-life (54). Zilucoplan, a macrocyclic peptide, works similarly but shows activity against eculizumab-resistant C5 protein variants (55). While C5 inhibition blocks terminal complement activation, it does not fully prevent upstream activity in the complement activation cascade, allowing continued deposition of C3 fragments and immune-mediated damage in some patients. This is particularly evident in disorders such as paroxysmal nocturnal hemoglobinuria (PNH), where residual extravascular hemolysis (typically in the liver and spleen) persists despite C5 blockade (56, 57). As a result, targeting C3 offers a broader approach by intercepting the cascade earlier and preventing both amplification and generation of downstream effectors like C3a, C5a, C3b/iC3b-mediated opsonization and MAC-induced cellular damage. Clinical evidence in PNH showed that C3 inhibition can more effectively control disease activity in cases where C5-targeted therapies fall short (58). For example, C3 inhibition can prevent both intravascular and extravascular hemolysis, and may provide a more comprehensive disease control in complement-mediated hemolytic disorders.

Rather than inhibiting the entire complement system, selectively targeting one or two of the pathways has emerged as promising therapeutic targets in immune-mediated disorders with improved safety. Molecules [e.g., plasma derived C1-INH (59)] and antibodies [e.g., sutimlimab (60) and ANX005 (61)] targeting at the CP offers selective modulation of complement activation in GBS and NMOSD (62–64).

Several inhibitors targeting the lectin pathway or both the classical and the lectin pathways are under active development. Narsoplimab (65), a humanized monoclonal antibody, selectively inhibits the lectin pathway effector MASP-2 without affecting the classical or alternative pathways, and has demonstrated clinical efficacy in hematopoietic stem cell transplantation-associated thrombotic microangiopathy (HSCT-TMA) (66). To modulate both the classical and lectin pathways, C2-targeting agents such as empasiprubart (formerly ARGX-117) (67) have been developed. Empasiprubart binds to the C2b fragment and is being investigated in multifocal motor neuropathy (MMN), a disease characterized by antibody-mediated complement activation at the nodes of Ranvier (57, 68). More recently, a nanobody-based inhibitor, Nab1B10, has been developed to target the C2a protease domain, exhibiting enhanced inhibitory potency compared to empasiprubart in preclinical models (69).

The alternative pathway has also gained attraction as a therapeutic focus, with Factor B, Factor D, and MASP-3 inhibitors showing promise in PNH (70) and lupus nephritis (71), along with age-related macular degeneration, a neurodegenerative retinal condition (72, 73). Although the trial that targets Factor D did not meet efficacy endpoints in geographic atrophy (GA) secondary to age-related macular degeneration (73), it demonstrated the feasibility of targeting alternative pathway proteins in retina. Together, these agents illustrate the growing interest in expanding the therapeutic reach beyond traditional C3 and C5 inhibition.

In addition to central and pathway-specific interventions, targeting complement-derived effector molecules such as the anaphylatoxins C3a and C5a has emerged as a promising strategy to modulate inflammation while preserving upstream immune surveillance. As mentioned earlier, these small peptides are potent pro-inflammatory mediators in the pathophysiology of numerous autoimmune, infectious, and neurodegenerative diseases. Among them, the C5a–C5aR1 axis has received particular attention due to its involvement in leukocyte recruitment, blood–brain barrier disruption, and neuroinflammation. Avacopan, the first oral C5aR1 inhibitor approved for anti-neutrophil cytoplasmic antibody (ANCA)-associated vasculitis, has paved the way for additional agents such as ACT-1014-6,470, ALS-205, and avdoralimab, which are currently under development for ANCA-associated vasculitis, post-traumatic epilepsy, and severe infections such as COVID-19 (74–77). Vilobelimab selectively inhibits C5a, but it’s *in vivo* specificity has been questioned—particularly in light of the high dosing regimen (800 mg) (78–80). Considering the naturally low circulating level of C5a, such high therapeutic doses raise the possibility of cross-reactivity with intact C5, which may partly account for its pharmacological effects. Beyond C5a and its receptor, the terminal pathway components C6 and C7 have also emerged as potential intervention points to block MAC (81, 82) and may be especially relevant in diseases where MAC-driven damage is a key pathogenic mechanism, such as AQP4 + NMOSD and autoimmune neuropathies.

### 4.2 Integrating complement regulators with targeted therapeutic strategies

In addition to inhibiting complement activation/effector proteins, supplementing and correcting the deficiency of complement regulators is another promising therapeutic strategy. For instance, exogenous FH delivery has been shown to restore complement homeostasis in FH-deficient mice (83). Engineered constructs such as mini-FH containing regulatory domains (CCP 1–5), binding domains (CCP 19–20), and CR2-FH, which uses CR2 to localize the therapeutic payload to complement activation sites, improve pharmacokinetics and tissue targeting (84). In murine models of collagen antibody-induced arthritis (85) and choroidal neovascularization (86), CR2-FH showed targeted inhibition of complement activation. More recently, AAV-mediated delivery of CR2-FH has been tested as a gene therapy for choroidal neovascularization, setting the stage for potential applications in retinal neurodegeneration and CNS demyelinating disorders (87).

Other approaches fuse antibodies against complement fragments, like C3d (88), with regulatory domains (FH or CR1) to provide localized complement inhibition (88–90). These approaches harness natural complement inhibitors and direct them precisely to inflamed tissues, enhancing therapeutic efficacy while minimizing systemic immunosuppression. Groups also attempt to conjugate CD55 with an antibody that recognizes the alpha subunit of the acetylcholine receptor. This targeted approach seems to improve the clinical score in experimentally acquired MG rat models (91). Chimeric fusion proteins combining regulatory or localization domains from CD55, FH, and CR1 further enhance regulation by accelerating convertase decay, promoting Factor I–mediated inactivation, and selectively targeting inflamed tissues (92).

## 5 Complement in PNS disorders

Complement activation represents a critical pathogenic element in numerous autoimmune PNS disorders, including MG, chronic inflammatory demyelinating polyneuropathy (CIDP), GBS, MMN, and dermatomyositis (DM). The susceptibility of the PNS to complement-mediated immune attack is increased by the lack of a protective barrier equivalent to the blood–brain barrier in the CNS. This section provides an overview of the complement system’s role in many autoimmune PNS conditions and uses current and emerging complement-targeting therapies to highlight disease pathophysiology. Complement-targeting therapies are summarized in Table 2.

**Table 2:** Current and emerging complement-targeted therapies for PNS disorders.

Agent	NM disorder	Clinical evidence
Eculizumab	myasthenia gravis	REGAIN – Phase 3 study in treatment-refractory AChR-Ab+ patients with gMG (*n* = 125) and its OLE study. Significant MG-ADL and QMG scores reduction; rapid and sustained improvement in ocular, bulbar, respiratory, and limb muscle strength (133, 239, 240). Dosage: 900 mg IV weekly for 4 weeks (initial dose), then 1,200 mg IV every 2 weeks (maintenance dose).
	Guillain-Barre syndrome	Two Phase 2 (*n* = 34, 7), and one Phase 3 (*n* = 57) trials: IVIg + placebo versus IVIg + Eculizumab, patients with GBS disability score 3 or more. Eculizumab tolerated, primary end point (predefined reduction in GBS disability score) not met. One Phase 2 trial showed trend for improved motor function in severe GBS subgroup (97–99).
	Multifocal motor neuropathy	One open label study (*n* = 13), tolerated, Only marginal benefit, no objective measurable improvements (111).
Ravulizumab	Myasthenia gravis	CHAMPION-MG – Phase 3 study in AChR-Ab+ patients with gMG (*n* = 175) and its OLE study. Significant, rapid and sustained MG-ADL and QMG scores reduction (134, 241). Dosage: 2400–3,000 mg IV (initial dose), then 3,000–3,600 mg IV every 8 weeks (maintenance dose). BW dose regimen (<60 kg, 60–100 kg, >60 kg).
Ziluclopan	Myasthenia gravis	RAISE – Phase 3 study in AChR-Ab+ patients with gMG (*n* = 174), and its OLE study (ongoing). Significant reduction in MG-ADL score (135). Interim analysis of OLE- sustained improvements till Week 120 for pooled zilucoplan 0.3 mg/kg patients (242). Dosage 0.3 mg/kg BW daily SC injections.
	Immune-mediated necrotizing myopathy	Phase 2 trial (*N* = 27), IMNM patients with HMGCR/SRP antibodies. No clinical improvement or significant change in creatinine kinase level between treatment and placebo group (154).
Tanruprubart (AXN005)	Guillain-Barre syndrome	Phase 3 trial (*N* = 241); patients with GBS Disability score 3–5. Patients treated with 30 mg/kg AXN005 achieved primary endpoint (GBS-disability score trichotomy at Week 8) vs. placebo; showed faster time to walk independently & less time on ventilation. Patients treated with 75 mg/kg AXN005 did not achieve primary endpoint as compared to placebo (100).
Pozelimab/ Cemdisiran	Myasthenia gravis	Phase 3 study, AChR-Ab+ patients with GMG (NCT05070858), ongoing.
	Sporadic inclusion body myositis	Single center study (NCT06479863), ongoing.
Gefurulimab (ALXN1720)	Myasthenia gravis	Phase 3 study- PREVAIL: AchR Ab+ gMG adult patients (*n* = 260). Clinically meaningful improvement in MG-ADL and QMG scores observed early during treatment and sustained through week 26. Similar incident of adverse events between treatment and placebo group (136).
Vemircopan (ALXN2050)	Myasthenia gravis	Phase 2 study, oral administration, (NCT05218096). primary efficacy end point not met; study terminated.
Claseprubart (DNTH103)	Myasthenia gravis	Phase 2 study- MaGic: AchR Ab + gMG patients (*n* = 65). Rapid, statistically significant and clinically meaningful improvements in MG-ADL and QMG scores in patients treated with 300 mg or 600 mg Claseprubart at Week 1 and at Week 13 (243).
	Chronic inflammatory demyelinating polyneuropathy	Phase 3 study (NCT06858579), ongoing.
	Multifocal motor neuropathy	Phase 2 study (NCT06537999), ongoing.
Riliprubart	Chronic inflammatory demyelinating polyneuropathy	Phase 2 open-label trial: CIDP patients divided into 3 groups: Standard of care (SOC- IVIg/corticosteroids) treated (*n* = 25), SOC- refractory (*n* = 18) and SOC- naïve (*n* = 12). After switching treatment to Riliprubart, improvement (≥1-point change in adjusted INCAT disability score) at week 24 seen in 44% SOC- treated, 50% SOC- refractory and 75% SOC- naïve patients. Clinically meaningful improvements were seen across multiple measures (104). Phase 3 study (NCT06290128, NCT06290141), ongoing
Empasiprubart (ARGX-117)	Multifocal motor neuropathy	Phase 2 study (*N* = 27): empasiprubart showed reduction in the risk of IVIg retreatment, improvement in grip/muscle strength, improvement in activity/disability levels (114). Phase 3 study (NCT06742190), ongoing.
	Dermatomyositis	Phase 2 study (NCT06284954), ongoing.

AChR, acetylcholine receptor; GMG, generalized myasthenia gravis; OLE, open label extension; MG-ADL, Myasthenia Gravis activity of daily living score; QMG, quantitative myasthenia gravis score, FDA, food and drug administration, EMA, European medicine agency.

### 5.1 Immune mediated neuropathies

#### 5.1.1 Guillain-Barré syndrome

Guillain-Barré syndrome encompasses a spectrum of acute autoimmune polyradiculoneuropathies primarily triggered by antecedent infections through molecular mimicry. Pathogenic autoantibodies of IgG1/IgG3 subclass, generated against microbial antigens (e.g., *campylobacter jejuni* lipo-oligosaccharides), cross-react with specific peripheral nerve gangliosides, such as GM1 and GD1a in the axonal form (acute motor axonal neuropathy (AMAN)) or myelin proteins/glycolipids in the demyelinating form (acute inflammatory demyelinating polyradiculoneuropathy (AIDP)) (93). Antibody binding triggers the CP and MAC formation, which leads ultimately to demyelination (in AIDP) or axonal degeneration (in AMAN). Pathological studies confirm deposition of activated complement components, including MAC, at sites of nerve damage in GBS patients (94, 95).

Intravenous immunoglobulin (IVIg) is an established treatment for patients exhibiting significant neurological disability for both CNS and PNS disorders. IVIg targets the immune system by multiple mechanisms, with complement inhibition being the predominant (96). Clinical trials targeting complement activation in GBS have yielded inconsistent results. Studies with eculizumab, when added to standard IVIg therapy, did not demonstrate significant clinical improvement over IVIg alone in Phase II and III trials (97–99). However, recent results from a Phase III study employing ANX005 (Tanruprubart), an anti-C1q antibody, showed significant clinical benefits in GBS patients (100). One of the reasons for the difference in efficacy seems to be from the site of action of these agents. C1q inhibition by Tanruprubart locks the CP at its origin, preventing the generation of not only MAC and C5a, but also the upstream anaphylatoxin C3a and the opsonin C3b. If C3-mediated inflammation and opsonization play a significant role in the pathogenesis of GBS (particularly in AIDP), C1q inhibition might offer a broader therapeutic benefit when compared to selective C5 inhibition by eculizumab (101).

#### 5.1.2 Chronic inflammatory demyelinating polyradiculoneuropathy

CIDP represents a spectrum of acquired immune-mediated PNS disorders characterized by progressive or relapsing–remitting demyelination. In a significant portion of CIDP cases mediated by IgG1/IgG3 subclasses, the complement system is a major effector pathway. Antibody binding to nerve targets initiates the CP via C1q leading to MAC formation and myelin breakdown (102). Studies have shown that levels of complement activation products C3a, C5a, and soluble MAC in the serum and the CSF remained unchanged in individuals who responded to IVIg therapy, which suggests other immunomodulatory mechanisms of IVIg in addition to complement inhibition (103). Preliminary results of a Phase II trial of Riliprubart in 25 CIDP patients showed favorable benefit–risk profiles and clinical improvement (104). A Phase III trial is ongoing for another C1s inhibitor, DNTH103 (NCT06858579), and is planned for the C2 inhibitor Empasiprubart (NCT06920004).

#### 5.1.3 Multifocal motor neuropathy

Multifocal motor neuropathy is an autoimmune disorder almost exclusively affecting motor nerves (105). Around 30–48% of patients with MMN have antibodies against GM1, a crucial ganglioside found in nerve myelin, particularly at the node of Ranvier (106). Anti-GM1 antibodies have been shown to activate complement and cause MAC deposition at the node of Ranvier in animal models, and complement activity seems correlating with MMN severity (105, 107–110).

Eculizumab was tested in an open label study in 13 MMN patients for 14 weeks (10 of whom were concomitantly receiving IVIg). The use of eculizumab did not change IVIg dosing, but there was some improvement in patient-rated subjective scores and clinical/electrophysiological measurements (111). TNT005 (Riliprubart), an investigational humanized C1s monoclonal antibody, was able to reverse complement deposition and functional deficits in a human-on-chip model of MMN (112). One study showed that IgM antibodies from MMN patients could bind motor neurons and activate complement cascade in an iPSC-derived motor neuron model. The study also showed efficacy of empasiprubart, a C2 inhibitory antibody, in blocking complement activation (113). Interim results of a Phase II trial in MMN patients showed that empasiprubart was well tolerated, reduced the risk of IVIg retreatment by 91% compared to placebo, and improved grip/muscle strength (114). A Phase III trial is ongoing (115).

#### 5.1.4 Myelin-associated glycoprotein neuropathy

Anti-Myelin-Associated Glycoprotein (MAG) IgM paraprotein-related neuropathy (anti-MAG PN) is a common chronic demyelinating neuropathy causing progressive imbalance, ataxia, sensory issues, and tremor (116, 117). Strong evidence implicates the role of anti-MAG antibodies, which bind to sulfoglucuronyl glycosphingolipid in peripheral nerves. In anti-MAG neuropathy, IgM and complement (including MAC) deposition on nerve myelin occurs, causing structural damage such as myelin widening and axonal atrophy. Several studies have found a correlation between the amount of IgM and complement deposition and the extent of myelin morphological changes (118, 119). While complement activation is observed, studies have not consistently linked anti-MAG antibody levels or complement activation directly to clinical disease severity. Therefore, current evidence does not provide strong support using complement inhibitors for this condition (120, 121).

### 5.2 Myasthenia gravis

**Figure 2:**
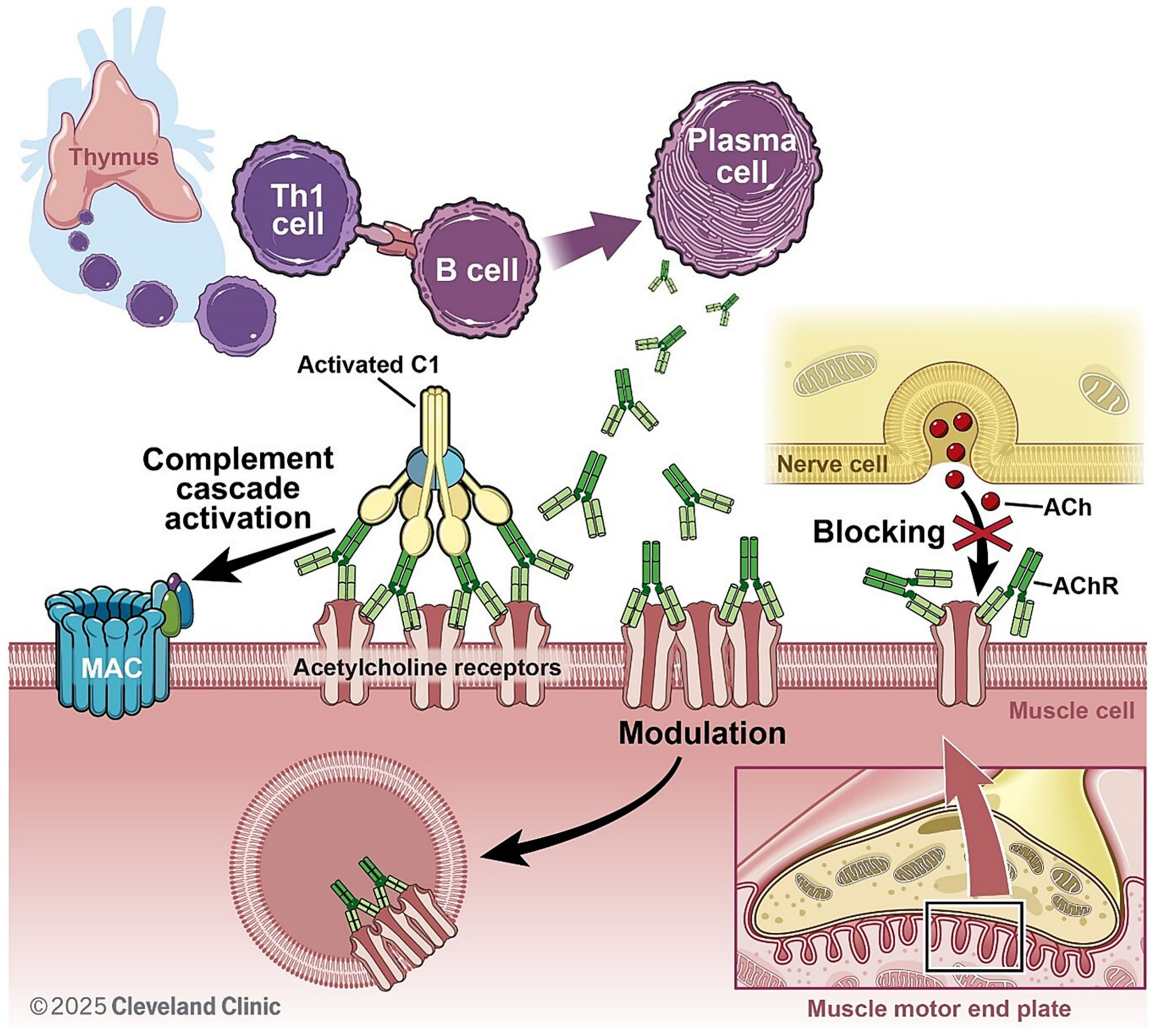
In AchR + MG, autoantibodies (produced by B cells with support from Th1 cells) are mainly of IgG1 subclass and activate complement cascade after binding to post synaptic acetylcholine receptors. This ultimately leads to MAC formation, pore formation on post synaptic membrane and internalization of AchR, reducing efficacy of neuromuscular junction transmission.

Myasthenia Gravis is an autoimmune disorder characterized by impaired neuromuscular transmission due to autoantibody-mediated dysfunction at the neuromuscular junction (NMJ) (Figure 2). Approximately 80% of MG patients have antibodies against acetylcholine receptor (AChR). Of the remaining 20% of MG patients, antibodies directed against muscle-specific kinase (MuSK) and low-density lipoprotein receptor-related protein- 4 (LRP4) were detected in the majority of patients (122).

In AChR, as well as LRP4, antibody-positive MG complement activation is a key pathogenic mechanism contributing to synaptic dysfunction. Pathogenic IgG1 and IgG3 autoantibodies trigger the classical complement cascade by engaging C1q, ultimately leading to opsonization, immune complex deposition, and the formation of MAC. This process culminates in reduced efficiency of neuromuscular transmission (123–126).

Research in the 1950s hinted at complement’s role in MG due to altered protein levels in patient sera (127–129). Subsequently, complement components (i.e., C3a and MAC) were observed directly at the NMJ of MG patients (130, 131). Furthermore, MG patient sera could induce complement-mediated destruction of cultured muscle cells. More recently, lower levels of properdin, a complement positive regulator, have been linked to more severe MG (132).

Complement mediated therapies have become established care in patients with AChR antibody positive MG. In 2017, eculizumab became the first complement inhibitor to gain regulatory approval for the therapy of AChR antibody positive generalized MG (gMG) on the basis of the REGAIN trial results (133). Based on positive results from the subsequent CHAMPION (134) and RAISE (135) trials, Ravulizumab and Zilucoplan were also approved as treatment of AChR positive gMG patients. Topline results from a recently completed trial accessing efficacy and safety profile of gefurulimab in AchR ab positive gMG patients showed significant and sustained improvement in MG ADL and Mg WMG scores (136). Trial outcomes, dosages, and emerging agents in closed and on-going trials are summarized in Table 2.

It should be noted that MuSK antibodies are mostly of the IgG4 subtype, which does not activate complement. Thus, complement inhibition therapy is ineffective in MuSK antibody-positive MG (137, 138).

### 5.3 Idiopathic inflammatory myopathies

#### 5.3.1 Dermatomyositis

Dermatomyositis is a systemic disease targeting skeletal muscle and skin (139). Approximately 60% of patients have myositis specific antibodies (i.e., anti-MDA-5, -NXP-2, −Mi-2, -TIF1-*γ*, -SAE-1) (140). An essential feature in the immunopathogenesis of classic DM is a complement-mediated microvasculopathy in the muscle, leading to capillary dropout, muscle ischemia, and anaphylatoxin-mediated muscle fiber damage (139, 141–145).

IVIg has been used as an effective treatment for DM (146–148). Novel complement-targeting agents have not demonstrated clear efficacy in the treatment of DM in clinical trials. A few case reports have shown efficacy of eculizimab in refractory dermatomyositis (149). A Phase II trial of ravulizumab for DM was terminated due to lack of efficacy (NCT04999020). A Phase II study has been initiated for empasiprubart, a C2 inhibitor, for the treatment of DM (NCT06284954). Ruxoprubart is a selective blocker of the alternative complement pathway that targets Factor B, leaving the CP intact. A Phase II efficacy trial of Ruxoprubart (NM8074) has been initiated for DM.

#### 5.3.2 Immune-mediated necrotizing myopathy

Immune-mediated necrotizing myopathy (IMNM) features significant muscle fiber damage with sparse inflammation, differing from DM and polymyositis. IMNM is linked to signal recognition particle or 3-hydroxy-3-methylglutarylcoenzyme A reductase autoantibodies. In IMNM, the complement system directly attacks muscle fibers. Pathogenic IgG autoantibodies bind to antigens on the muscle fiber surface (sarcolemma), activate the classical complement pathway, and lead to MAC formation and, finally, muscle fiber necrosis (150–153).

A Phase II trial investigated zilucoplan in adult IMNM assuming complement activation being the primary driver for disease pathophysiology. Although the drug effectively and safely inhibited the complement pathway, it failed to improve muscle enzyme (CK) levels or clinical symptoms. This lack of efficacy may contradict the prevailing theory that complement-driven MAC deposition is the main driver of IMNM. These results, supported by preclinical models (where therapeutic administration of zilucoplan following disease onset failed to significantly restore muscle strength in humanized mice model), suggest that IMNM autoantibodies may cause muscle damage through complement-independent mechanisms (i.e., antibody mediated muscle fiber dysfunction) (154, 155).

#### 5.3.3 Inclusion body myositis

Inclusion body myositis (IBM) is a common acquired muscle disease in individuals over 50 years of age. Autoantibodies against cytoplasmic 5′-nucleotidase (cN1A) have been found in 35–80% of IBM patients, however, their presence does not correlate clearly with disease severity or survival (156, 157). Recent immunological findings have intensified the debate regarding inflammation as the primary pathogenic driver in IBM. Passive transfer experiments have demonstrated that serum from anti-NT5C1A seropositive IBM patients can induce disease-specific pathology in both myotube cultures and mouse models, supporting an antibody-mediated inflammatory component (158). One study has shown upregulation of MAC (C5b-9) in muscle biopsies from 17 of 20 patients with IBM (159). These findings suggest a potential role of complement activation in IBM.

To date, no immunosuppressive or complement-targeting therapies have demonstrated efficacy in the treatment of IBM. A clinical trial investigating the efficacy and safety of Pozelimab and Cemdisiran combination therapy in IBM is ongoing (NCT06479863).

### 5.4 Immune check point-related neuromuscular disorders

Neurologic immune-related adverse events develop in approximately 2–4% of patients with neuromuscular conditions receiving immune checkpoint inhibitors (ICIs), which constitutes the majority of cases (160). Proposed mechanisms for immune-related adverse events involve primarily T-cell dysfunction, potentially including enhanced T-cell activity against antigens shared by tumors and healthy tissues, coupled with elevated inflammatory cytokines (161, 162). There is also some evidence suggesting a role for B cells, indicated by the development of autoantibodies after ICI treatment (163). Additionally, the complement system appears to be involved. Enhanced complement-mediated inflammation may occur due to direct antibody binding [e.g., CTLA4-IgG (164)] or due to increased levels of pre-existing antibodies. Although not studied extensively, one report documented complement deposition around muscle fibers and capillaries in a patient with ICI-related myasthenia-myositis-myocarditis-overlap syndrome (165). Furthermore, successful treatment of ICI-associated MG with eculizumab and ravulizumab has been reported, suggesting the relevance of complement activation in these ICI related disorders (133, 164, 165).

## 6 Complement activation and treatment in the CNS

Complement activation is involved in the pathogenesis of multiple autoimmune CNS disorders and serves as a key driver of pathogenicity in AQP4 + NMOSD. Complement inhibitors have been explored as treatment options in multiple PNS disorders and are approved and undergoing clinical trials for AQP4 + NMOSD in the CNS. This section provides an overview of the complement system’s role in many CNS conditions, focusing on proposed pathophysiological mechanisms. Current and emerging complement-targeting therapies are summarized briefly in Table 3.

**Table 3:** Current and emerging complement-targeted therapies for CNS disorders.

Agent	CNS disorder	Clinical evidence
Eculizumab	NMOSD relapse prevention	PREVENT – Phase 3 placebo-controlled study in AQP4 + patients with EDSS≤7 and relapses within past 2 years (*n* = 143). Adjunctive IST allowed but not rituximab. Significant reduction in relapses with eculizumab, 3% vs. 43%, HR = 0.06. In the OLE, 96% were relapse-free at 4 years (53, 244). FDA approved for treatment of AQP4 NMOSD. Dose: 900 mg IV weekly for 4 weeks (initial dose), then 1,200 mg IV every 2 weeks (maintenance dose).
	NMOSD acute attack	EASE-NMO Phase 2 trial in AQP4 + patients presenting within 10 days of acute symptom onset. Addition of eculizumab to IVMP, not yet recruiting (NCT06673394).
Ravulizumab	NMOSD relapse prevention	CHAMPION-NMOSD – Phase 3 placebo-controlled study in AQP4 + patients with EDSS≤7 and relapses within past 2 years (*n* = 105). Adjunctive IST allowed but not rituximab. Significant reduction in relapses with ravulizumab, 0% vs. 43%, HR = 0.014. In the OLE, no relapses occurred during median follow up of 138.4 weeks. Significant reduction in relapses with ravulizumab, 0% vs. 43%, HR = 0.014. In the OLE, no relapses occurred during median follow up of 138.4 weeks (176, 245). FDA approved for treatment of AQP4 + NMOSD. Dose: 2400–3,000 mg IV (initial dose), then 3,000–3,600 mg IV every 8 weeks (maintenance dose). BW dose regimen (<60 kg, 60–100 kg, >60 kg).
Cinryze	NMOSD acute attack	Phase 1b open-label trial in patients with AQP4 + or – NMOSD presenting with acute transverse myelitis or optic neuritis (*n* = 10). Addition of C1-INH to steroids in acute relapse was associated with decreased EDSS at discharge and 30-day follow-up. Phase 1b open-label trial in patients with AQP4 + or – NMOSD presenting with acute transverse myelitis or optic neuritis (*n* = 10). Addition of C1-INH to steroids in acute relapse was safe but efficacy data is limited (62).

### 6.1 Aquaporin-4 IgG-positive neuromyelitis optica spectrum disorder

Aquaporin-4 IgG-positive Neuromyelitis Optica Spectrum Disorder is an inflammatory CNS disorder defined by the presence of pathogenic autoantibodies to the water channel AQP4 (166). Core clinical inflammatory syndromes occur in areas with highest AQP4 expression and blood brain barrier (BBB) permeability, including the optic nerve, spinal cord, area postrema, diencephalon, and cerebrum.

Unknown immune tolerance checkpoint defects enable AQP4 IgG to be produced by autoreactive plasmablasts differentiated from immature B cells in the peripheral blood (167–169). Peripheral AQP4 IgG breaches the BBB by unclear mechanisms and binds AQP4 located on the end feet of astrocytes lining the BBB. The pathogenic M23 isoform of AQP4 assembles orthogonal arrays of particles, which facilitate AQP4 IgG clustering on the cell membrane and enable optimal C1q binding. AQP4 IgG binding triggers an immunologic cascade resulting in further BBB disruption, complement activation, astrocyte injury, secondary demyelination, and neuronal cell death (170). Destruction is mediated by complement-mediated cytotoxicity via MAC generation, as well as additional inflammatory mechanisms generated through production of the anaphylatoxin C5a (171). Lesions characteristically demonstrate extensive demyelination, edema, axonal injury, perivascular complement deposition, and necrosis (27, 170, 172, 173). Multiple *in vivo* and *ex vivo* studies have shown that both AQP4 IgG and complement are required to reproduce typical AQP4 + NMOSD lesions (27, 173, 174). Additionally, C5a levels are increased in the CSF of AQP4 + NMOSD compared to MS and other CNS disorders and correlate with severity of exacerbations (171, 175).

The role of complement in NMSOD pathophysiology was further confirmed by two Phase III clinical trials, PREVENT and CHAMPION-NMOSD, showing that both eculizumab and ravulizumab significantly decrease relapses in patients with AQP4 + NMOSD (53, 176). In 2019, eculizumab became the first FDA-approved maintenance treatment for AQP4 + NMOSD followed by ravulizumab in 2024. A Phase Ib trial showed that treating acute NMOSD relapses with the C1-esterase inhibitor Cinryze and steroids was safe but efficacy data was limited (62). The Phase II trial, EASE-NMO (NCT06673394), aims to explore the addition of eculizumab to steroids in acute relapses is ongoing.

### 6.2 Multiple sclerosis

Multiple sclerosis is a chronic CNS disorder characterized by inflammation, demyelination, and neuronal degeneration (177). Evidence is growing for the role of B cells, antibodies, and the innate immune system in pathogenesis, supported by the success of B-cell-depleting treatments and Bruton’s tyrosine kinase inhibitors (i.e., tolebrutinib) (178, 179). Additionally, evidence exists to support varying degrees of complement activation in MS. Histopathological studies showed evidence of complement deposition along the MS disease spectrum with complement becoming increasingly prominent as the disease progresses (180, 181). In progressive MS, complement markers are present in chronic active lesions, chronic inactive lesions, and normal appearing white matter despite the absence of other inflammatory components. Inflammation relies on innate (i.e., macrophages and CNS-resident microglia) rather than adaptive mechanisms (182–184).

Although there is evidence of complement involvement in MS pathogenesis, the source and the trigger of complement are unclear. Systemic complement was initially thought to enter the CNS through BBB disruption similar to other CNS autoimmune conditions. However, complement can also be produced by CNS cells, and compartmentalized intrinsic activation of complement by degenerating neurons independent of cell-mediated demyelination may contribute to pathogenesis, including synaptic pruning, in progressive diseases such as MS. In addition, complement may be activated initially to promote the clearance of degradation products and other debris, but the ineffective clearance of complement-targeted products by senescent microglia may lead to aberrant activation with demyelination and synapse loss (185, 186).

There is currently no clinical trial data investigating the use of complement inhibitors in MS. Preclinical data has shown complement inhibitors have some benefit in experimental autoimmune encephalomyelitis mouse models (187–189). In case reports, relapsing and progressive MS patients treated with eculizumab for other indications continued to demonstrate a clinical and radiographical deterioration of their MS (190, 191). The complex pathophysiology around MS, along with the fact that it is not an antibody-associated disorder, makes any conclusive statements difficult. Whether complement activation represents a primary pathogenesis or is secondary to other inflammatory events remains uncertain.

### 6.3 Myelin oligodendrocyte glycoprotein antibody-associated disease

Myelin oligodendrocyte glycoprotein antibody-associated disease (MOGAD) is defined by the presence of myelin oligodendrocyte glycoprotein (MOG)-IgG. However, MOG-IgG has been seen in a small proportion of patients with other neurologic diseases and is less specific at low titers, raising questions on its pathogenicity, especially in contrast to AQP + NMOSD (192–196). MOGAD histopathology is characterized by inflammatory demyelination, particularly in perivascular regions, without astrocyte destruction. Areas of demyelination show varying loss of MOG staining and mainly consist of macrophages and T-cells with CD4 + dominancy but also show some evidence of complement-mediated destruction (195).

Similar to AQP4 IgG, MOG-IgG is predominantly IgG1 subtype and capable of fixing complement (197). However, the extent of complement activation and overall role of complement in MOGAD pathophysiology is not completely understood. MOG protein constitutes a relatively minor component of the myelin sheath, making it difficult for antibodies to cluster. MOG-IgG also requires bivalent binding, which poorly binds C1q, diminishing subsequent complement activation (198). Compared to patients with AQP4 + NMOSD, patients with MOGAD have significantly lower levels of MAC but similarly increased levels of the anaphylatoxins C3a and C5a in CSF, suggesting that complement activation is present, but may generate destruction through mechanisms other than MAC-mediated cytotoxicity (199). Additionally, a recent study evaluated serum and CSF complement levels and showed that combinations of various complement factors have diagnostic and prognostic value in MOGAD patients. Combination of serum levels of C3a, C4a, and the C3a/C3 ratio were able to discriminate MOGAD from MS and NMOSD with MOGAD demonstrating higher levels of C3a and C4a compared to other pathologies. In the CSF, lower C4 levels were associated with a higher number of relapses during follow up, and a high C4a/4 ratio was associated with increased risk of second relapse during the first year and shorter time to second relapse. Further, elevated baseline levels of the MAC SC5b9 in the CSF were associated with increased risk of disability on the Expanded Disability Status Scale, suggesting complement inhibition could have therapeutic benefit in this disease (200).

However, MRI T2 lesions in MOGAD show complete resolution more frequently compared to AQP4 + NMOSD and MS, which raises questions regarding the underlying pathophysiology as complement activation has been shown to be highly destructive in AQP4 + NMOSD (201). Overall, given varying levels of complement activity demonstrated in multiple studies, the role of complement activation in MOGAD pathophysiology is unclear. Although IVIg has been shown to be effective in preventing MOGAD attacks, there are currently no ongoing clinical trials investigating complement inhibitors (202, 203).

### 6.4 Autoimmune encephalitis & related disorders

The diagnosis of antibody associated autoimmune encephalitis (AE) is suggested by the subacute onset of cognitive or neuropsychiatric symptoms along with a CNS inflammation usually detected on MRI or CSF studies (204). Clinical phenotypes can be further defined by the presence of a corresponding antibody, which can target intracellular or cell surface/synaptic receptors leading to diverse pathophysiology. Complement has been investigated in the pathogenesis of AE with variable results.

The role of complement is most studied in AE related to N-methyl D-aspartate (NMDA) antibodies. NMDA antibodies are IgG1 subclass and able to activate complement *in vitro*. However, when examined at autopsy, cases of NMDA encephalitis showed prominent microgliosis and deposits of IgG with rare inflammatory infiltrates and no complement deposits, suggesting complement may not play a role *in vivo* (205–208).

A study of 52 patients with glycine antibody positive patients found that glycine antibodies are mainly of the IgG1 subclass and were capable of activating complement *in vitro* (209). Histology from patients with contactin-associated protein-like 2 (CASPR2) antibodies who underwent temporal lobe resections for seizures showed the presence of T cells, B cells, and microglia, but complement deposition was identified in only a few neurons despite strong leakage of IgG through the BBB (210). In patients with glial fibrillary acidic protein (GFAP) antibodies, histopathology showed prominent immunoreactivity of astrocytes with the complement activation product C4d, but did not detect the deposition of IgG, C1q, and terminal complement complex on astrocytes (211). Another study examined hippocampal tissue from patients with glutamic acid decarboxylase (GAD) antibodies and found increased transcriptional levels for genes encoding complement proteins. Hippocampal C3d deposition was visualized in 5 of 7 patients (212). Although these studies suggest complement may play a role in autoimmune encephalitis, more investigation is needed to determine the specific mechanisms for each antibody.

IVIg is used routinely to treat a variety of AEs with varying efficacies, likely supporting a role for complement activation (213, 214). The best evidence for IVIg is within the GAD antibody–spectrum disorders. One double-blind, placebo-controlled trial with IVIg in stiff person syndrome showed significant improvements in mobility and spasms, which translated into improved activities of daily living. This is one of the few disorders that has clear, prospective evidence (Level 1a) for IVIg usage (215, 216). In case reports of glycine antibody-associated stiff person syndrome, one patient had significant improvement on eculizumab, while another patient did not experience improvement and was quickly taken off eculizumab due to the associated complication of meningitis (217).

### 6.5 Primary angiitis of the central nervous system

Primary angiitis of the CNS (PACNS) is a rare form of vasculitis limited to the brain, spinal cord, and leptomeninges that leads frequently to cerebral ischemia and hemorrhage. Histopathology shows granulomatous inflammation, lymphocytic infiltration, and acute necrotizing vasculitis, but the exact pathogenesis remains unknown (218). The role of complement activation has been explored in patients with PACNS with conflicting results. A study evaluating the CSF from 8 biopsy-proven cases of PACNS suggests that the complement cascade is a significant feature of the disease. Complement proteins in the alternative pathway and terminal complement were significantly upregulated, and the MAC inhibitor CD59 was significantly downregulated in PACNS compared to noninflammatory controls (219, 220). Another study evaluating the serum and CSF in 20 patients with PACNS found that levels of complement activation including components specific to the classical and the alternative pathways were unchanged in PACNS patients when compared to those from noninflammatory controls (221). There are currently no ongoing clinical trials investigating complement inhibitors in PACNS.

## 7 Future directions

### 7.1 Balancing between complement activation control and host defense

While all pathway inhibition at C3/C5 levels can control complement overactivation effectively, long-term complement inhibition increases the susceptibility to infections, particularly those caused by certain bacteria, such as meningococcal species. This risk is highlighted by the FDA black box warnings issued to all complement inhibitors (222), requiring vaccinations prior to initiating these complement-targeting therapies to mitigate infection risks. The latest Center for Disease Control recommendation for adult patients with complement deficiencies including the usage of complement inhibitor therapies includes a series of vaccinations for meningococcal serogroups A, C, W, and Y along with serogroup B. The complete series takes 6 months to complete and vaccination for meningococcal must remain up to date while on therapy. Antimicrobial prophylaxis is required, but the duration of antimicrobial prophylaxis is currently at clinicians’ discretion. At our centers, we typically will also include pneumococcal 20-valent and haemophilus b conjugate vaccines if not previously administered.

Breakthrough infections can still occur even after full vaccination, especially in immunocompromised patients. For example, the Meningococcal B vaccine targets *Neisseria meningitidis* serogroup B (MenB), a leading cause of bacterial meningitis across all age groups in many industrialized countries. However, it is not fully protective, with a range between approximately 76 and 83% (223, 224). Real-world data and post-marketing surveillance have also revealed additional non-meningococcal infections, including pneumonia, bacteremia, sepsis and septic shock, urinary tract infections, staphylococcal infections, and opportunistic viral infections (225). These findings underscore the need for ongoing infection monitoring, booster vaccinations, and patient education, particularly in vulnerable populations (e.g., those with neuro-immunological comorbidities).

Beyond concerns of acute safety events, prolonged complement suppression may impair immune surveillance, a function critical to the clearance of apoptotic and early neoplastic cells (226). Recent studies have revealed that complement plays a dual role in cancer (227). On one hand, complement enhances antibody-mediated cytotoxicity through CP activation and MAC formation. On the other hand, aberrant complement activation in the tumor microenvironment can foster tumor progression by sustaining chronic inflammation, promoting angiogenesis, and recruiting myeloid-derived suppressor cells (MDSCs) that suppresses T cell responses. In neurological cancers and metastases affecting the CNS, complement components such as C3a and C5a may also disrupt the blood–CSF barrier, further facilitating tumor spreading (32, 228). This complex, context-dependent interplay highlights the importance of maintaining a tenuous balance of proper complement activation to avoid tipping from protective immunosurveillance toward tumor-supportive inflammation.

Unlike pan-complement blockade, which eliminates the formation of opsonins (C3b, iC3b) and MAC, pathway-specific inhibition allows complement activity to remain partially intact. This targeted approach may reduce the risk of severe infections, minimize immune dysregulation and off-target tissue damage, and avoid undesired immunosuppression–factors especially important for pediatric, elderly, or immunocompromised patients (229). In diseases where autoantibody-mediated activation of the CP drives pathology, such as AQP4 + NMOSD, GBS, and MG, selective inhibition of C1s or C1q can reduce immune complex-driven inflammation without compromising the alternative or lectin pathways. Sutimlimab (229) and ANX005 (230) exemplify this strategy, demonstrating the ability to dampen neuroinflammation leading to meaningful clinical improvement while minimizing typical safety concerns associated with broader complement blockade.

The alternative pathway serves as an amplification loop for all complement activation, but in many diseases its overactivity alone is sufficient to result in tissue damage. Targeted inhibitors of Factor B (Iptacopan) and Factor D (Danicopan, Vemircopan) can attenuate this amplification precisely. This may be especially beneficial in chronic neurological disorders, where localized complement dysregulation contributes to glial activation, synapse loss (231), and axon degeneration (232).

### 7.2 Precision-targeted and combination therapeutic strategies

Next,-generation complement therapies are moving toward context-sensitive modulation and synergistic combination regimen to maximize efficacy while minimizing systemic immunosuppression. For instance, CR2-targeted inhibitors as mentioned in section 4.2 are engineered to localize specifically to sites of complement activation by binding deposited C3b/iC3b fragments, ensuring focused inhibition at diseased tissues.

Complement inhibitors can also be deployed in combination with other immunomodulatory agents to enhance therapeutic outcomes, particularly in antibody-mediated neurological disorders, as exemplified by recent strategies in oncology and ophthalmology. A notable clinical-stage example is the Phase II trial (NCT04919629) evaluating APL-2, a C3 inhibitor, in combination with pembrolizumab (anti-PD-1) and/or bevacizumab (anti-VEGF). This combination strategy aims to reduce immunosuppressive C5a signaling in the tumor microenvironment, where C5a recruits myeloid-derived suppressor cells and inhibits CD8^+^ T cell function (233), thereby potentiating the efficacy of ICIs. In a separate domain, bispecific fusion proteins, efdamrofusp alfa (234) and KNP-301 (235, 236), which bind both C3b and VEGF, demonstrated selective inhibition of the alternative pathway and anti-angiogenic effects in preclinical models of neovascular and dry age-related macular degeneration.

Together, these examples highlight the promise of dual-targeted or combination-based strategies that modulate both complement and other disease-relevant pathways as a blueprint for treating complex autoimmune neurological disorders, where complement is only one component of a multifaceted pathological network.

Gene therapies are also being explored to restore function in cells lacking surface complement regulators. For instance, GT005 (237), an AAV-based therapy encoding Factor I, and HMR59, encoding a soluble form of CD59 (NCT03144999), are being evaluated for their ability to reinstate complement regulation in target tissues in age-related macular degeneration. Additionally, nano-vesicular delivery systems incorporating GPI-anchored complement regulators offer a novel means of protection against complement-mediated cell lysis. In studies using biomimetic proteolipid vesicles, human membrane proteins were embedded in synthetic phospholipid bilayers to reconstitute missing GPI-anchored regulators on the surface of complement-vulnerable cells. These vesicles significantly enhanced resistance to complement attack in both healthy and patient-derived blood cells (238). Both healthy and PNH peripheral blood mononuclear blood cells treated with these biomimetic lipid vesicles exhibited increased resistance to complement-mediated lysis.

## 8 Conclusion

Over the past decade, complement-related therapeutic advances, including those targeting central components such as C3 and C5 or specific pathways, have transformed the clinical landscape for many immune-mediated neurological disorders diseases. Ongoing therapeutic strategies into context-specific complement regulation, effector-targeted strategies, and gene-based delivery platforms promises to further refine these approaches. Yet, challenges remain to balance complement activation with therapeutic suppression, to manage patient heterogeneity, and to identify biomarkers that predict complement-driven pathology. As our mechanistic understanding deepens and precision therapies evolve, we will develop superior therapies with various modifying strategies that are both safer and more effective.
